# The effect of magnesium added to bupivacaine for arthroscopy: a meta-analysis of randomized controlled trials

**DOI:** 10.1186/s13018-021-02609-w

**Published:** 2021-10-10

**Authors:** Weineng Xiang, Lin Jiang, Langtao Shi, Chengming Jiang, Yun Zhou, Chunhua Yang

**Affiliations:** Department of Spin Surgery, The First Hospital of Changsha, NO.311 Yingpan Road, Kaifu District, Changsha City, Hunan China

**Keywords:** magnesium sulphate, bupivacaine, arthroscopy, randomized controlled trials, meta-analysis

## Abstract

**Introduction:**

The analgesic efficacy of magnesium sulphate added to bupivacaine for arthroscopy remains controversial. We conduct a systematic review and meta-analysis to explore the efficacy of magnesium sulphate in combination with bupivacaine for arthroscopy.

**Methods:**

We searched PubMed, EMbase, Web of science, EBSCO, and Cochrane library databases through July 2020 for randomized controlled trials (RCTs) assessing the effect of magnesium sulphate plus bupivacaine versus bupivacaine for arthroscopy. This meta-analysis is performed using the random-effect model.

**Results:**

Six RCTs were included in the meta-analysis. Overall, compared with bupivacaine for arthroscopy, combination analgesia using magnesium plus bupivacaine was associated with significantly prolonged duration of analgesia (SMD=0.93; 95% CI=0.27 to 1.60; P=0.006) and first time to analgesic requirement (SMD=196.57; 95% CI=13.90 to 379.24; P=0.03), reduced pain scores (SMD=-1.71; 95% CI=-2.96 to -0.46; P=0.007) and analgesic consumption (SMD=-1.04; 95% CI=-1.49 to -0.60; P<0.00001), but showed no remarkable influence on nausea or vomiting (OR=1.54; 95% CI=0.60 to 3.97; P=0.37).

**Conclusions:**

Magnesium sulphate added to bupivacaine may significantly improve the analgesic efficacy for arthroscopy.

## Introduction

Arthroscopic surgeries are commonly used for various diagnostic and therapeutic purposes [1–4]. However, they may result in serious postoperative pain in some patients. Adequate pain relief is crucial for early mobilization, rehabilitation and discharge of patients [5–8]. Several strategies have been developed to achieve optimal analgesia, and include systemic narcotics, nonsteroidal anti-inflammatory drugs, neuraxial analgesia (spinal/epidural), peripheral nerve blocks, intravenous patient-controlled analgesia, periarticular (e.g. subcutaneous, intra-articular) infiltration with local anesthetics [9, 10].

Many local anesthetics were used for arthroscopy, such as lignocaine, bupivacaine, ropivacaine, morphine, fentanyl, sufentanil, pethidine, clonidine, dexmedetomidine, magnesium, ketorolac, tramadol, and neostigmine et al [11–13]. However, there are still some limitations such as short duration of action, restricted availability in the wards, and need for stringent monitoring for any side effects. Optimal modality can provide excellent analgesia with minimal side effects, lower opioid use and enhance rehabilitation. Bupivacaine was commonly used for the analgesia of arthroscopy [12, 14]. Magnesium sulfate acts as an NMDA (N-Methyl-D-Aspartate) receptor antagonist, and was found to improve the pain management after arthroscopic meniscectomy when added to levobupivacaine [15].

Several studies reported the magnesium sulfate plus bupivacaine versus bupivacaine for arthroscopic surgery, but their efficacy has not been well established [15–18]. With accumulating evidence, we therefore perform a systematic review and meta-analysis of RCTs to compare the analgesic efficacy of magnesium sulfate plus bupivacaine versus bupivacaine for arthroscopy.

## Materials and methods

Ethical approval and patient consent were not required because this was a systematic review and meta-analysis of previously published studies. The systematic review and meta-analysis were conducted and reported in adherence to PRISMA (Preferred Reporting Items for Systematic Reviews and Meta-Analyses) [19].

### Search strategy and study selection

Two investigators have independently searched the following databases (inception to July 2020): PubMed, EMbase, Web of science, EBSCO, and Cochrane library databases. The electronic search strategy was conducted using the following keywords: magnesium, and bupivacaine, and arthroscopy or arthroscopic surgery. We also check the reference lists of the screened full-text studies to identify other potentially eligible trials.

The inclusive selection criteria were as follows: (i) patients underwent knee arthroscopy; (ii) intervention treatments were magnesium sulphate plus bupivacaine versus bupivacaine; (iii) study design was RCT.

### Data extraction and outcome measures

We have extracted the following information: author, number of patients, age, female, weight, duration of surgery and detail methods in each group etc. Data were extracted independently by two investigators, and discrepancies were resolved by consensus. We also contacted the corresponding author to obtain the data when necessary.

The primary outcomes were duration of analgesia and pain scores. Secondary outcomes included first time to analgesic requirement, analgesic consumption, nausea and vomiting.

### Quality assessment in individual studies

Methodological quality of the included studies was independently evaluated using the modified Jadad scale [20]. There was 3 items for Jadad scale: randomization (0-2 points), blinding (0-2 points), dropouts and withdrawals (0-1 points). The score of Jadad Scale varied from 0 to 5 points. An article with Jadad score≤2 was considered to be of low quality. If the Jadad score≥3, the study was thought to be of high quality [21].

### Statistical analysis

We estimate the standard mean difference (SMD) with 95% confidence interval (CI) for continuous outcomes (duration of analgesia, pain scores, first time to analgesic requirement, and analgesic consumption) and odd ratio (OR) with 95%CI for dichotomous outcomes (nausea and vomiting). The random-effects model was used regardless of heterogeneity. Heterogeneity was reported using the I^2^ statistic, and I^2^ > 50% indicated significant heterogeneity [22]. Whenever significant heterogeneity was present, we searched for potential sources of heterogeneity via omitting one study in turn for the meta-analysis or performing subgroup analysis. All statistical analyses were performed using Review Manager Version 5.3 (The Cochrane Collaboration, Software Update, Oxford, UK).

## Results

### Literature search, study characteristics and quality assessment

**Fig. 1:**
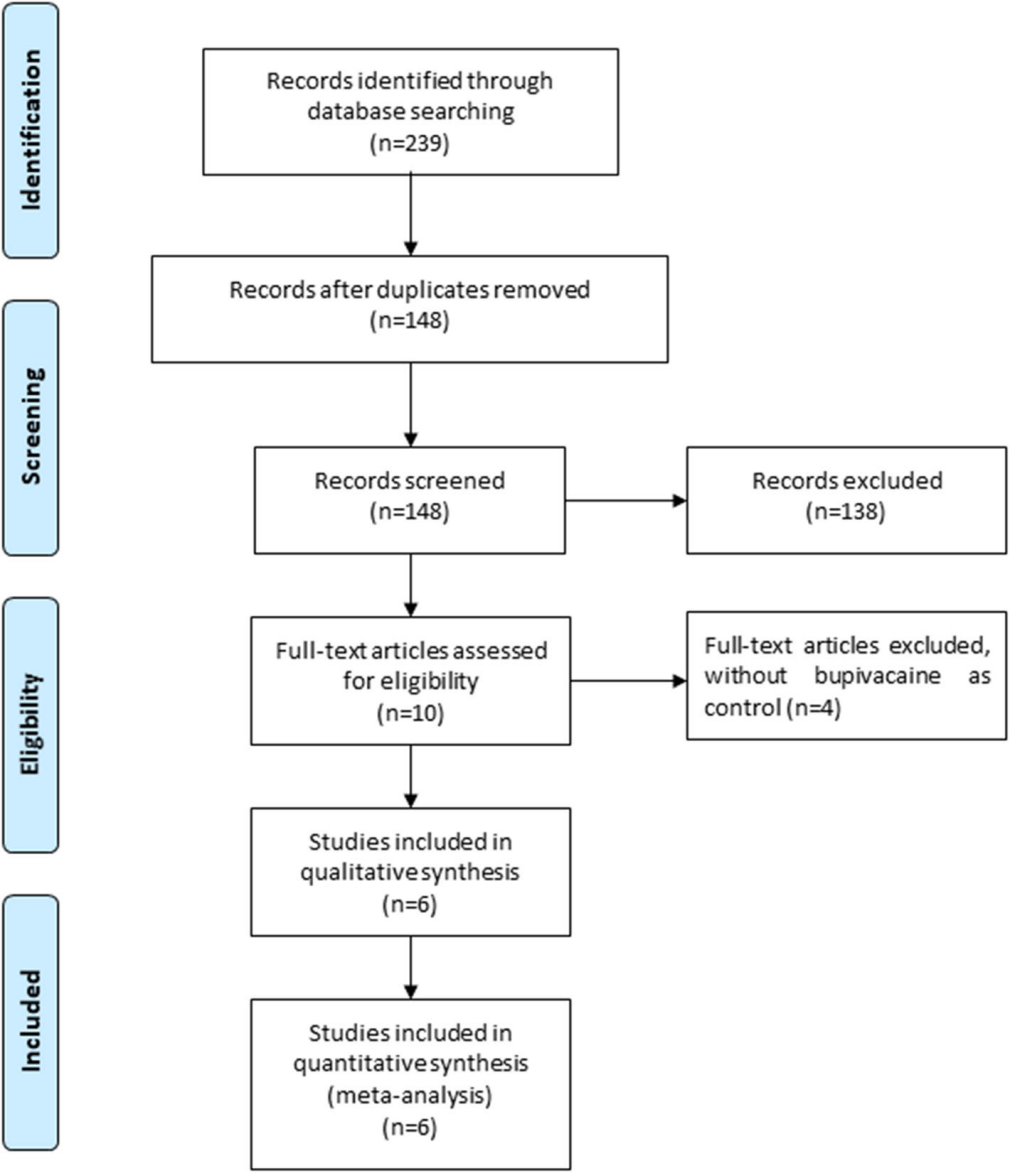
Flow diagram of study searching and selection process

A detailed flowchart of the search and selection results was shown in Fig. 1. 239 potentially relevant articles were identified initially. 91 duplicates and 138 papers after checking the titles/abstracts were excluded. Six RCTs were ultimately included in the meta-analysis [15–18, 23, 24].

The baseline characteristics of the six eligible RCTs in the meta-analysis were summarized in Table 1. The six studies were published between 2008 and 2018, and sample sizes ranged from 36 to 60 with a total of 312. Among the six included RCTs, there were knee arthroscopy [15, 16, 18, 23, 24] and shoulder arthroscopy [17]. The doses of magnesium sulphate ranged from 50 mg to 1.5 g. Two studies reported duration of analgesia [16, 17], two studies reported pain scores [15, 17], two studies reported first time to analgesic requirement [18, 23], four studies reported analgesic consumption [15, 18, 23, 24], two studies reported nausea and vomiting [17, 23]. Jada scores ranged from 3 to 5, and thus these six included generally had high quality.

**Table 1:** Characteristics of included studies

NO.	Author	Magnesium sulfate group						Control group						Jada scores
		Number	Age (years)	Female (n)	Weight (kg)	Duration of surgery (min)	Methods	Number	Age (years)	Female (n)	Weight (kg)	Duration of surgery (min)	Methods	
1	Devi 2018	18	33.44±10.87	5	67.67±14.11	-	intraarticular 20ml, 0.25% bupivacaine with 10 mg/kg of magnesium sulfate for knee arthroscopy	18	37.22±13.36	8	67.39±8.33	-	20 ml of 0.25% bupivacaine	3
2	Kızılcık 2017	32	43.06±13.19	10	-	-	50 mg (5 ml) of levobupivacain and an additional 1.5 g magnesium sulfate (10 ml) intraarticularly for arthroscopic meniscectomy	32	40.06 ± 9.24	8	7-	-	100 mg (10 ml) of levobupivacaine intraarticularly	4
3	Lee 2012	28	60±8	10	67±11	90±22	interscalene nerve block was performed with 0.5% bupivacaine 20 mL with epinephrine (1:200,000) plus either 10% magnesium sulphate 2 mL for arthroscopic rotator cuff repair	30	56±8	13	71±19	98±45	interscalene nerve block was performed with 0.5% bupivacaine 20 mL with epi- nephrine (1:200,000) plus normal saline 2 mL	5
4	Farouk 2009	20	36± 6	2	74±7	40±8	20 ml 0.25% bupivacaine and magnesium 150 mg for arthroscopic knee surgery	20	35 ± 7	4	72 ± 8	41 ± 8	20 ml 0.25% bupivacaine	4
5	Dayioğlu 2009	30	41.2 ± 15.3	11	74.2 ± 12.1	56.3±20.8	50 mg magnesium sulfate (3 ml) following 6 mg bupivacaine 0.5% plus 10 μ g fentanyl intrathecally for knee arthroscopy	30	38.7 ± 14.4	12	75.5 ± 8.1	52.9 ± 18.2	3 ml of preservative-free 0.9% NaCl following 6 mg bupivacaine 0.5% plus 10 μ g fentanyl intrathecally	4
6	Elsharnouby 2008	27	40±11	1	82±12	-	bupivacaine 0.25% and 1 g of magnesium sulfate in 20 mL for arthroscopic knee surgery	27	45±9	2	79±11	-	0.25% (20 mL) bupivacaine	4

### Primary outcomes: duration of analgesia and pain scores

**Fig. 2:**
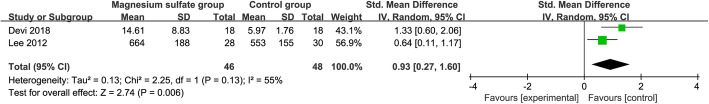
Forest plot for the meta-analysis of duration of analgesia

**Fig. 3:**
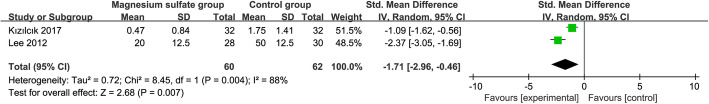
Forest plot for the meta-analysis of pain scores

These outcome data were analyzed with the random-effects model, and compared to bupivacaine for arthroscopy, combination analgesia using magnesium plus bupivacaine was associated with significantly prolonged duration of analgesia (SMD=0.93; 95% CI=0.27 to 1.60; P=0.006) with significant heterogeneity among the studies (I^2^=55%, heterogeneity P=0.13) (Fig. 2) and reduced pain scores (SMD=-1.71; 95% CI=-2.96 to -0.46; P=0.007) with significant heterogeneity among the studies (I^2^=88%, heterogeneity P=0.004) (Fig. 3).

### Sensitivity analysis

Significant heterogeneity was observed for the primary outcomes. There were only two studies included, and thus we did not perform the sensitivity analysis by omitting one study in turn or the subgroup analysis.

### Secondary outcomes

**Fig. 4:**
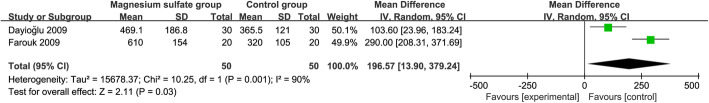
Forest plot for the meta-analysis of first time to analgesic requirement

**Fig. 5:**
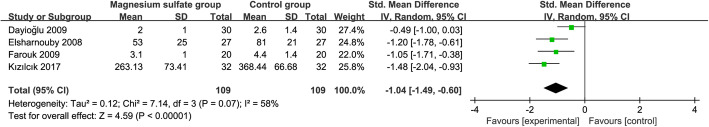
Forest plot for the meta-analysis of analgesic consumption

**Fig. 6:**
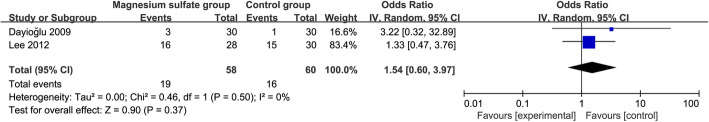
Forest plot for the meta-analysis of nausea and vomiting

In comparison with bupivacaine for arthroscopy, combination analgesia could substantially increase the first time to analgesic requirement (SMD=196.57; 95% CI=13.90 to 379.24; P=0.03; Fig. 4), and reduce the analgesic consumption (SMD=-1.04; 95% CI=-1.49 to -0.60; P<0.00001; Fig. 5), but revealed no obvious effect on nausea or vomiting (OR=1.54; 95% CI=0.60 to 3.97; P=0.37; Fig. 6).

## Discussion

Surgical excision and resection in arthroscopy produce postoperative pain because of irritation of free nerve ending of synovial tissue, anterior fat pad, and joint capsule [25–27]. Preoperative evaluation, appropriate intraoperative management and early postoperative mobilization are crucial for the anesthesia for ambulatory surgery needs good [28, 29]. Multimodal analgesia has become a promising approach for postoperative pain relief. For instance, intraarticular analgesic agents are used as a simple and cost-effective approach via acting on peripheral receptors and providing analgesia locally with minimal systemic side effects. The local tissue binding to receptors can be increased in order to enhance the analgesic effect [30].

Local anesthetics such as bupivacaine may exert chondrotoxicity by reducing chondrocyte viability in arthroscopy surgeries [31]. The incidence of chondrolysis following local administration of bupivacaine seems to be low or possibly underreported [32]. Bupivacaine is combined with adjuvants (e.g. opioids, Mg, tramadol, clonidine and dexmedetomidine) to provide prolonged postoperative analgesia and reduce the dose of analgesics. N-methyl D-aspartate (NMDA) receptors widely exist centrally and peripherally in the joints, muscles, and skin. They account for central nociceptive transmission, modulation, and sensitization of acute pain states [33]. Mg can serve as an efficacious adjunct, therefore enhancing postoperative analgesia when used by intravenous, intraarticular and epidural routes [14, 33, 34]. Its antinociceptive effect is produced by blocking NMDA receptors and decreasing the entry of extracellular calcium into cells, thereby exhibiting chondrocyte protective effect [24].

The main findings of this meta-analysis suggested that combination analgesia using magnesium plus bupivacaine could significantly prolong the duration of analgesia and first time to analgesic requirement, reduce pain scores and analgesic consumption than only bupivacaine for arthroscopic surgeries, but there was no increase in nausea and vomiting after magnesium plus bupivacaine intervention. Significant heterogeneity remained when performing the sensitivity analysis, and several factors may account for it. Firstly, different types and operation procedures can produce different levels of pain intensity, which may affect the pooling results. Secondly, the doses of magnesium sulphate ranged from 50 mg to 1.5 g, and produce different promotion to pain relief. Thirdly, the routes of local analgesics included intraarticular and spinal anesthesia.

This meta-analysis has several potential limitations. Firstly, our analysis is based on six RCTs, and all of them have a relatively small sample size (n<100). These may lead to overestimation of the treatment effect in smaller trials. More RCTs with large sample size should be conducted to explore this issue. Next, different doses and routes of drugs may contribute to the significant heterogeneity. Finally, different types and operation procedures can produce different levels of pain intensity, which may produce some effect on the results.

## Conclusions

Combination analgesia using magnesium plus bupivacaine may provide better analgesic efficacy for arthroscopy than as compared to only bupivacaine.

## Abbreviations

RCTs: Randomized controlled trials
MDs: Mean differences
CIs: Confidence intervals
RRs: risk ratios
